# Innovation of a Regulatory Mechanism Modulating Semi-determinate Stem Growth through Artificial Selection in Soybean

**DOI:** 10.1371/journal.pgen.1005818

**Published:** 2016-01-25

**Authors:** Yunfeng Liu, Dajian Zhang, Jieqing Ping, Shuai Li, Zhixiang Chen, Jianxin Ma

**Affiliations:** 1 Department of Agronomy, Purdue University, West Lafayette, Indiana, United States of America; 2 College of Life Sciences, Qingdao Agricultural University, Qiangdao, Shandong, China; 3 Department of Botany and Plant Pathology, Purdue University, West Lafayette, Indiana, United States of America; Peking University, CHINA

## Abstract

It has been demonstrated that *Terminal Flowering 1* (*TFL1*) in Arabidopsis and its functional orthologs in other plants specify indeterminate stem growth through their specific expression that represses floral identity genes in shoot apical meristems (SAMs), and that the loss-of-function mutations at these functional counterparts result in the transition of SAMs from the vegetative to reproductive state that is essential for initiation of terminal flowering and thus formation of determinate stems. However, little is known regarding how semi-determinate stems, which produce terminal racemes similar to those observed in determinate plants, are specified in any flowering plants. Here we show that semi-determinacy in soybean is modulated by transcriptional repression of *Dt1*, the functional ortholog of *TFL1*, in SAMs. Such repression is fulfilled by recently enabled spatiotemporal expression of *Dt2*, an ancestral form of the *APETALA1/FRUITFULL* orthologs, which encodes a MADS-box factor directly binding to the regulatory sequence of *Dt1*. In addition, *Dt2* triggers co-expression of the putative *SUPPRESSOR OF OVEREXPRESSION OF CONSTANS 1* (*GmSOC1*) in SAMs, where GmSOC1 interacts with Dt2, and also directly binds to the *Dt1* regulatory sequence. Heterologous expression of *Dt2* and *Dt1* in determinate (*tfl1*) *Arabidopsis* mutants enables creation of semi-determinacy, but the same forms of the two genes in the *tfl1* and *soc1* background produce indeterminate stems, suggesting that *Dt2* and *SOC1* both are essential for transcriptional repression of *Dt1*. Nevertheless, the expression of *Dt2* is unable to repress *TFL1* in Arabidopsis, further demonstrating the evolutionary novelty of the regulatory mechanism underlying stem growth in soybean.

## Author Summary

Similar to the “green revolution” semi-dwarf cereals, semi-determinate soybean varieties are lodging-resistant and particularly suitable for planting in high fertility and irrigated environments. Nevertheless, molecular mechanisms underlying semi-determinate stem growth have not been deciphered in any flowering plants. We demonstrate that semi-determinacy is originated from an innovation of spatiotemporal expression of an ancient MADS-box gene and consequent changes of spatiotemporal expression of its interacting genes in soybean, which occurred post-domestication of soybean and selected by breeding. The findings from this study not only provides new insights into the evolutionary novelty of molecular mechanisms regulating stem growth habit reshaped by artificial selection, but also exhibited potential application of such an innovative mechanism for molecular design of stem architecture in other crops towards enhanced adaptability and yield potential.

## Introduction

Stem growth habit is an important morphological and adaptation trait in flowering plants, which is primarily shaped by regulatory processes converting the vegetative shoot apical meristems (SAMs) that form leaves to the inflorescence meristems (IMs) and then floral meristems (FMs) that form flowers [1–3]. Such processes have been best studied in Arabidopsis [4, 5]. Upon floral induction by both environmental signals (e.g., day length, light quality, and temperature) and endogenous cues (e.g., age and hormone), the primary shoot meristems of Arabidopsis begin to produce the determinate IMs on its flanks, where the floral identity genes such as *LEAFY* (*LFY*) and *APETALA1* (*AP1*) are expressed to develop flowers [6–8]. However, the SAMs in the center of the stem tips sustain the indeterminate growth due to the spatial expression of a floral repressor *Terminal Flower1* (*TFL1*) [1, 9, 10], which represses the expression of *LFY* and *AP1* and thus prevents the formation of FMs [11–12]. As a result, the wild-type (*TFL1*) Arabidopsis produces the indeterminate apical stems that grow indefinitely. By contrast, *LFY* and *AP1* are expressed in the center of stem tips of the loss-of-function *tfl1* mutants to give rise to determinate growth, with terminal flowers on the stem tips [1, 8, 11, 13, 14]. In addition, both LFY and AP1 are able to bind to the *TFL1* locus to suppress its expression in floral meristems [15, 16].

Although the full functions of *TFL1* in Arabidopsis remain to be elucidated, it is documented that the putative orthologs of *TFL1* are widely conserved among diverse plant species including many leguminous and solanaceous species, and in particular, their roles as floral repressors, such as *Dt1* in soybean (*Glycine max*) [17, 18], *PvTFL1y* in common bean (*Phaseolus vulgaris*) [19], *Det* in pea (*Pisum sativum*) [20], *Sp* in tomato (*Solanum lycopersicum*) [21], and *CaSP* in peppers (*Capsicum annuum*) [22], in producing indeterminate stems are conserved. In general, the wild progenitor species of these individual crops and the majority of the cultivated varieties from these species exhibit indeterminate stem growth. Nevertheless, determinate growth habit in these crops was also selected through domestication or modern breeding, and adapted to specific eco-regions for agricultural production [23–25].

The determinate soybean varieties rose originally from independent human selections of four distinct single-nucleotide substitutions in the *Dt1* gene during soybean domestication from its wild progenitor *Glycine soja*, each of which led to a single amino acid change that resulted in a recessive *dt1* allele specifying determinate stem growth [17]. In general, determinate soybean cultivars have distinctly separate vegetative and reproductive stages and are relatively late maturing and grown in the southern eco-regions of both the United States and China. By contrast, the indeterminate cultivars have more overlap of vegetative growth with reproductive development, providing better adaptation to shorter growing season in the north [26]. In addition to these two major types of stem growth habit, semi-determinate cultivars, which produce stems with terminal racemes similar to those observed in determinate cultivars but show an intermediate phenotype have been developed through breeding in the past few decades and deployed for production in the north. While semi-determinate cultivars usually produce slightly fewer stem nodes than indeterminate cultivars, the former are somewhat shorter than the latter and thus provide some degree of lodging resistance that is desirable for production in the high fertility and irrigated environments [27], representing an alternative for enhancement of soybean yield potential, similar to that achieved by the “green revolution” gene in cereals.

Classic genetic analysis demonstrated that semi-determinacy in soybean is specified by a dominant allele, designated *Dt2*, in the *Dt1* genetic background [23]. As shown in S1 Table, the *dt2dt2*;*Dt1Dt1* and *Dt2Dt2*;*Dt1Dt1* genotypes produce indeterminate and semi-determinate plants respectively, whereas both the *dt2dt2;dt1dt1* and *Dt2Dt2;dt1dt1* genotypes produce determinate plants, indicating a recessive epistatic effect of *dt1* on the *Dt2/dt2* locus. Semi-determinate stem growth habit was also observed and genetically investigated in tomato [21, 28–30] and two other leguminous crops, pigeon pea (*Cajanus cajan*) [24] and chickpea (*Cicer arietinum*) [25]. However, unlike observed in soybean, semi-determinacy in tomato is specified by a recessive allele *sdt* in the recessive *sp* genetic background, and the dominant allele *Sp*, the functional equivalent of *TFL1/Dt1*, exhibits dominant epistatic effect on the *Sdt/sdt* locus [21, 28–30] (S1 Table). More intriguingly, the legume crops pigeon pea and chickpea, two close relatives of soybean, both show an inheritance pattern of stem growth habit and a digenic epistasis distinct from observed in soybean but similar to observed in tomato [24, 25]. A more recent study demonstrated that the genetic control of stem growth habit in pea is also distinct from observed in soybean [31] (S1 Table). These observations reflect the uniqueness and evolutionary novelty of genetic control of stem growth habit in soybean.

Recently, *Dt2* has been isolated by a map-based cloning approach using a segregating population derived from a high-yielding semi-determinate elite soybean cultivar NE3001 and a high-yielding indeterminate elite cultivar IA3023 [32] (S1 Fig). *Dt2* was demonstrated to be a dominant gain-of-function MADS-domain factor gene belonging to an *AP1*/SQUAMOSA subfamily that includes floral identity genes *AP1*, *CAULIFLOWER* (*CAL*), *FRUITFUL* (*FUL*) in Arabidopsis [33–35]. It was also found that the causative mutation that converting *dt2* into *Dt2* is located in the non-coding regulatory region of the gene. Quantitative real time-polymerase chain reaction (qRT-PCR) analysis revealed that *Dt2* is primarily expressed in the stem tips at vegetative 2 (V2) stage, when the first trifoliate leaflets at node 2 are fully expanded but the second trifoliate leaflets at node 3 are not yet unfolded. It was proposed that, at this stage, floral induction occurs in all meristems (apical and lateral), abruptly in the case of the determinants, less abruptly in the case of semi-determinants, but not in the terminal apical meristems in indeterminants, suggesting the essential role of *Dt2*, as a floral activator, in promoting terminal flowering with the presence of *Dt1*. However, except of the observed phenotypic epistasis, it is not yet known how this recently selected dominant gain-of-function *Dt2* allele interacts with *Dt1* and other genes to modulate the semi-determinate growth habit. Here, we report molecular dissection of the *Dt2*-mediated molecular mechanism regulating stem growth habit in soybean, with an emphasis on the evolutionary novelty of the regulatory pathways reshaped by artificial selection.

## Results

### The Expression *Dt1* is Repressed by the Expression of *Dt2*

**Fig 1:**
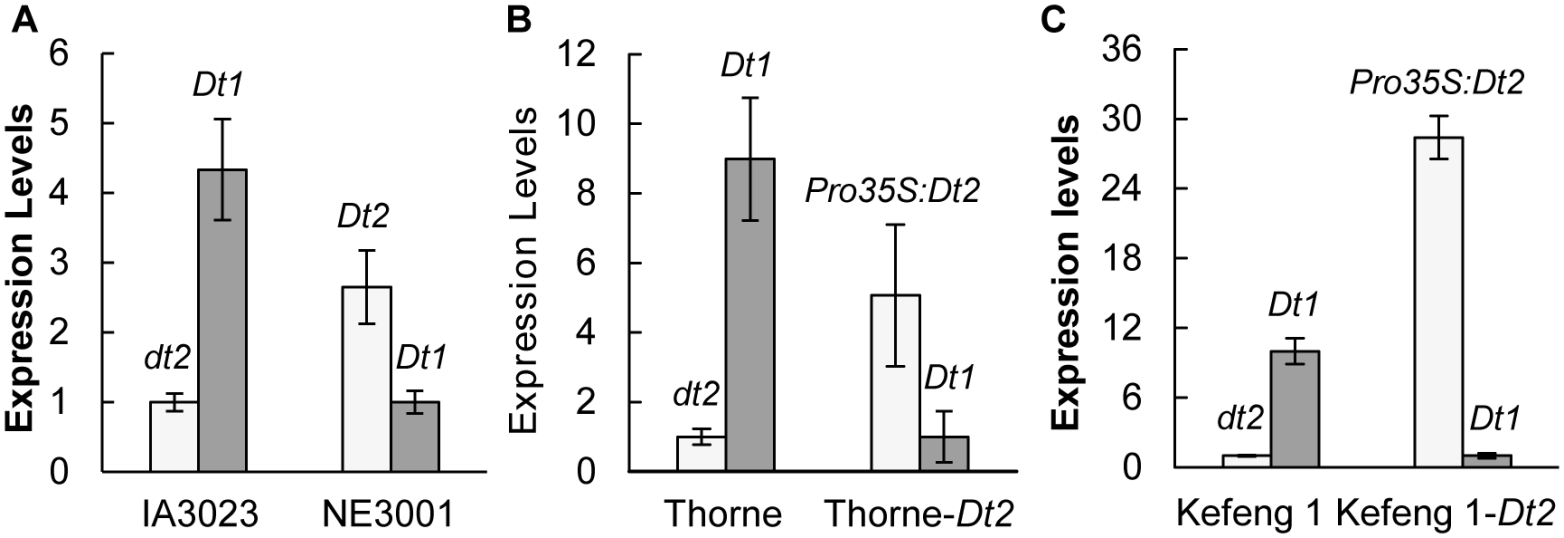
Association of *Dt1* repression with *Dt2* expression revealed by qRT-PCR. (**A**) Expression levels of *Dt1* and Dt2/dt2 in the stem tips of IA3023 and NE3001 at the V2 stage. Expression levels of *dt2* in IA3023 and *Dt1* in NE3001 were set as 1, and those of *Dt2* in NE3001 and *Dt1* in IA3023 were adjusted accordingly. (**B**) Expression levels of *Dt1* and *Pro35S*:*Dt2/dt2* in the stem tips of Thorne and Thorne transgenic line at the V2 stage. Expression levels of *dt2* in Thorne and *Dt1* in Thorne transgenic line were set as 1, and those of *Pro35S*:*Dt2* in the transgenic line and *Dt1* in Throne were adjusted accordingly. (**C**) Expression levels of *Dt1* and *Pro35S*:*Dt2/dt2* in non-transgenic and transgenic hairy roots of Kefeng 1. Expression levels of *dt2* in non-transgenic hairy roots and *Dt1* in transgenic hairy roots were set as 1, and those of *Pro35S*:*Dt2* in transgenic roots and *Dt1* in non-transgenic roots were adjusted accordingly. Values in the (**A**) and (**C**) are shown as mean δ standard errors of the means from three biological replicates, while values in the (**B**) are shown as mean δ standard errors of the means from three technical replicates.

The expression patterns of the *Dt1* and *Dt2* loci in the main stem tips of NE3001 (*Dt2Dt2;Dt1Dt1*) and IA3023 (*dt2dt2;Dt1Dt1*) have been previously examined by qRT-PCR [32] (Fig 1A). The expression level of either the *Dt2* or *dt2* allele was increased from the V0 (when the cotyledons at node 0 are fully extended but the unifoliate leaflets at node 1 are not yet unrolled) to V2 stages and then decreased at the V3 stage (when the second trifoliate leaflets are fully expanded but before the third trifoliate leaflets are still unrolled). By contrast, the expression level of *Dt1* in either the *Dt2* or *dt2* backgrounds was consistently reduced over these developmental stages. In addition, the expression level of *Dt1* in the *Dt2* background was lower than detected in the *dt2* background. Such an expression pattern, particularly at the V2 stage, together with the epistatic interaction between the two genes as deduced from the phenotypes [23], suggest that *Dt2* may be a transcriptional repressor of *Dt1*. However, because the apical meristems only made up a small portion of the main stem tips, the relative abundance of the transcripts from the two genes in apical meristems could not be precisely reflected by qRT-PCR analysis. Therefore, how the difference in levels of *Dt2* and *dt2* expression determines indeterminate or semi-determinate stems was not understood.

To further elucidate the effects of the *Dt2* expression on the transcription of *Dt1*, we analyzed the expressional changes of *Dt1* under ectopic expression of *Dt2* driven by the Cauliflower Mosaic Virus (CaMV) 35S promoter. As shown in Fig 1B, the expression level of *Dt1* in stem tips at the V2 stage was significantly reduced upon the ectopic expression of the transgene *Dt2* in the Throne (*dt2dt2;Dt1/Dt1*) genetic background, which resulted in an conversion from indeterminate stems to semi-determinate stems [32].

Because the expression level of *Dt1* is extremely low relative to that of *Dt2* or *dt2* in the stem tips [32], it is quite difficult to accurately determine the extent of repression of the *Dt1* expression. Previous work has demonstrated that *Dt1* is expressed at the highest level in soybean roots [17], where both the *Dt2* and *dt2* alleles are expressed at extremely low levels [32]. Thus, in the soybean root system, there appear to be little or no effects of the native *Dt2/dt2* locus on the expression of *Dt1*, making the system ideal for investigation of the effect of *Dt2* on the expression of *Dt1*. Using an indeterminate soybean cultivar Kefeng 1 (*dt2/dt2;Dt1/Dt1*), we generated the *Dt2* over-expression transgenic hairy roots. As shown in Fig 1C, the *Dt2* transgene driven by the 35S promoter in the transgenic hairy roots was expressed at a level ~30 times higher than the native *dt2*. By contrast, the expression level of the native *Dt1* was reduced ~10 times in the *Dt2* transgenic roots, indicating that the level of the *Dt2* expression is a key factor controlling *Dt1* expression.

### Dt2 Directly Binds to the Promoter Region of *Dt1*

**Fig 2:**
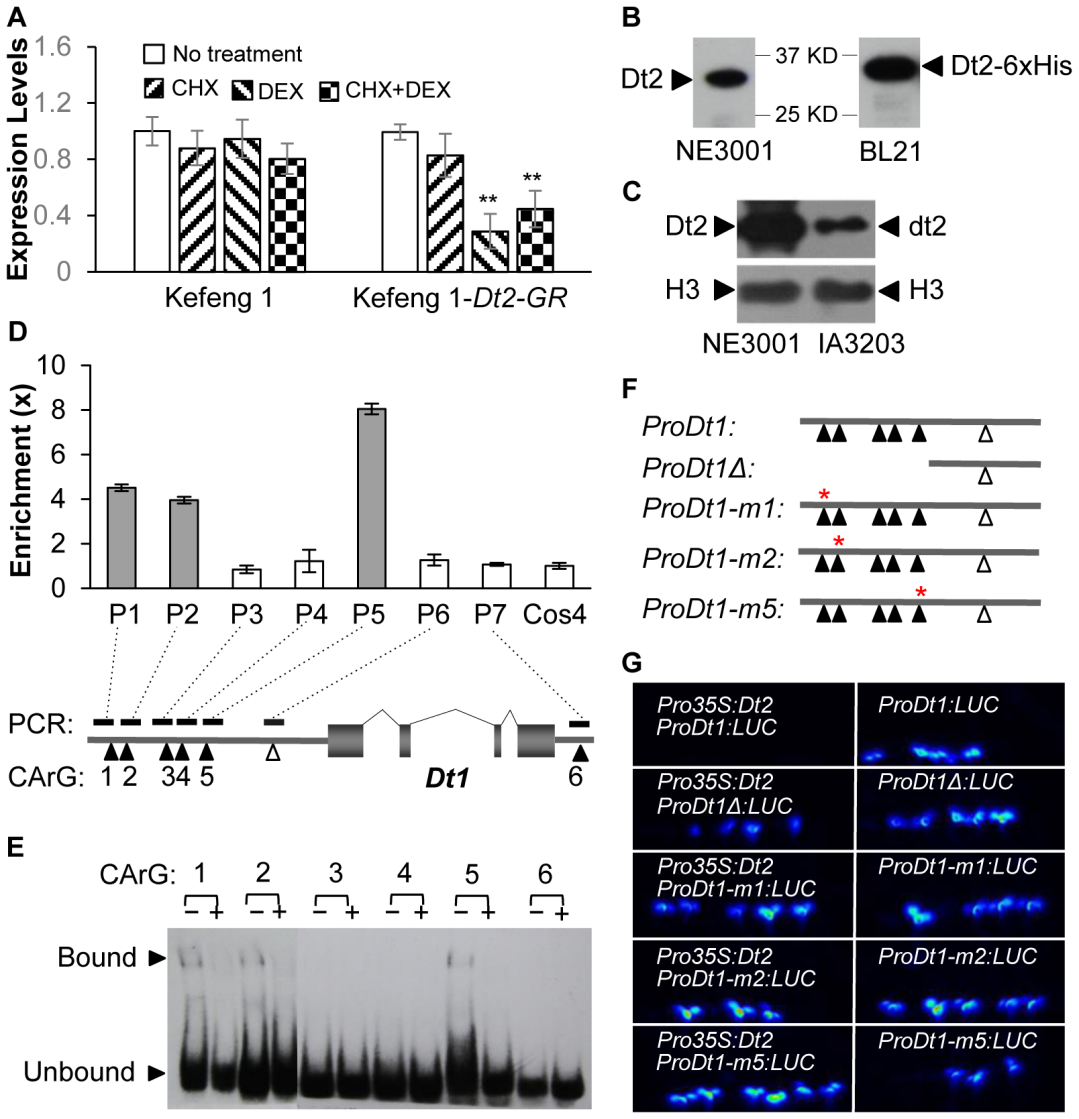
Binding of *Dt2* to the promoter region of *Dt1* repressing its expression. (**A**) Expression levels of *Dt1* in the *Dt2-GR* non-transgenic and transgenic roots of Kefeng 1 under different treatments. Expression level of *Dt1* in the untreated sample in each group was set as 1, and those of the other three treated samples were adjusted accordingly. Values are shown as mean δ standard errors of the means from three biological replicates. Doubled-asterisks indicate significant difference compared with DEX-treated samples (*t*-test, *p*<0.01). (**B**) Dt2 in the total proteins from the stem tips of NE3001 and Dt2-6×His fusion protein from *E*. *coli* detected by the anti-Dt2 antibody through Western blotting. (**C**) Levels of Dt2/dt2 and Histone H3 proteins from the stem tips of NE3001 and IA3023 detected by the anti-Dt2 and H3 antibody, respectively, through Western blotting. (**D**) Relative enrichment of fragments from the regulatory region of *Dt1* by anti-Dt2 detected by ChIP-PCR, using a fragment from *Cons4* as a control. Physical locations of the six fragments each harboring a putative CArG-box (solid triangles), a randomly chosen fragment without any putative CArG box (a triangle frame) are shown in the schematic diagram, in which the exons and introns of *Dt1* and its regulatory regions are represented by gray boxes, curve lines, and thick lines, respectively. The relative enrichment of the *Cons4* fragment was set as 1.0 and those of other fragments were adjusted accordingly. Values are shown as mean δ standard errors of the means from three biological replicates. (**E**) Dt2-binding sites in the regulatory region of *Dt1* validated by EMSA. The “+” and “−” indicate reactions with and without unlabeled competitor probes, respectively. Upper and lower arrows indicate probes bound and unbound by the Dt2-6×His fusion protein, respectively. (**F**) Diagrammatic illustration of truncated or mutated sequences of the Dt1 promoter, as shown in (**E**), which were used to express luciferase (LUC) as shown in (**G**). *ProDt1Δ* represents the *Dt1* promoter with deletion of a fragment containing the three CArG boxes bound by Dt2, whereas *ProDt1-m1*, *ProDt1-m2*, *ProDt1-m5* represent the *Dt1* promoter each with a 3–4 nucleotide mutation, indicated by an asterisk, within the 1^st^, 2^nd^, and 5^th^ CArG boxes, respectively. (**G**) Essential role of each of the three CArG-boxes in fulfilling the repression of *Dt1* transcription by Dt2 revealed by analysis of LUC expression in Arabidopsis.

Since Dt2 is a MADS-box domain transcription factor (TF) localized in nucleus [32], we wondered whether Dt2 could directly interact with the *Dt1* promoter to inhibit the transcription of *Dt1*. To this end, we created a fusion of the *Dt2* protein to the hormone-binding domain of the rat glucocorticoid receptor (GR), under the control of the constructive 35S promoter (*Pro35S*:*Dt2-GR*), and the construct was transformed into the hairy roots of the soybean cultivar Kefeng 1. To determine the effect of Dt2 activation on the expression of *Dt1* in the *Pro35S*:*Dt2-GR* transgenic roots, we treated the roots with the steroid hormone dexamethasone (DEX), the protein synthesis inhibitor cycloheximide (CHX) or both and then measured the changes of *Dt1* expression by qRT-PCR, using non-transgenic hairy roots as a control. As shown in Fig 2A, the level of the *Dt1* mRNA was reduced significantly after the treatment with DEX, indicating that DEX activation of the Dt2-GR fusion protein by nuclear translocation [13, 36] resulted in repression of *Dt1*. The level of *Dt1* mRNA was also reduced significantly in the presence of both DEX and CHX, but not reduced in the presence of CHX, which has been proven to be able to terminate de novo protein synthesis [13, 36], suggesting that Dt2 can transcriptionally repress *Dt1* directly without the requirement for protein synthesis.

As MADS box domains are generally able to bind to DNA sequences of high similarity to the motif CC[A/T]_6_GG termed the CArG-box [37], we first examined the *Dt1* sequence and its flanking sequences from NE3001 and identified five putative CArG-boxes within ~2kb upstream of the *Dt1* coding sequence (CDS) and one putative CArG-box at ~1.5kb position downstream of the CDS (Fig 2D). To determine whether Dt2 directly binds to the CArG-box sequences in the regulatory region of *Dt1*, we performed chromatin immunoprecipitation (ChIP). We first raised a Dt2-specific antibody, anti-Dt2 (Fig 2B) based on a highly unique peptide composed of 19 amino acids from Dt2 (S2 Fig), according to the soybean reference genome sequence [38], and its specificity was further indicated by a substantially higher level of the Dt2 protein detected in the stem tips of NE3001 than that of dt2 detected in IA3023 at the V2 stage (Fig 2C). We then used the anti-Dt2 antibody to enrich DNA fragments bound by Dt2 in NE3001 and then measured the relative enrichment by quantitative PCR (qPCR). As shown in Fig 2D, fragments containing the 1^st^, 2^nd^, and 5^th^ putative CArG-boxes, respectively, were enriched by >5–9 fold compared with the control DNA fragment amplified from the soybean ATP binding cassette transporter gene *Cons4* [39] in the same genome. By contrast, the 3^rd^ and 4^th^ putative CArG-boxes, and the 7^th^ one downstream of the *Dt1* gene were not enriched compared with the control, indicating that these three of the six CArG-boxes are recognized and bound by Dt2. These results were consistent with the observations from the electrophoretic mobility-shift assay (EMSA) analysis (Fig 2E), which reveals that only 1^st^, 2^nd^, and 5^th^ CArG-boxes can be bound by a Dt2-6×His fusion protein isolated from an *Escherichia coli* strain BL21 (Fig 2B). The essentiality of the three CArG boxes for the repression of *Dt1* transcription by Dt2 was further demonstrated by the observed ineffectiveness of the repression activity upon the truncation of the *Dt1* promoter region involving these CArG-boxes or point mutations within each of the three CArG boxes using a luciferase (LUC) as a reporter (Fig 2F and 2G). Together, these observations suggest that Dt2 functions as a repressor of *Dt1* expression by binding directly to the three CArG boxes in the *Dt1* promoter region through its MADS-box domain, and that all these three CArG boxes, bound by Dt2, are essential for repression of the activity of the *Dt1* promoter.

### Dt2 Directly Interacts with GmSOC1

**Fig 3:**
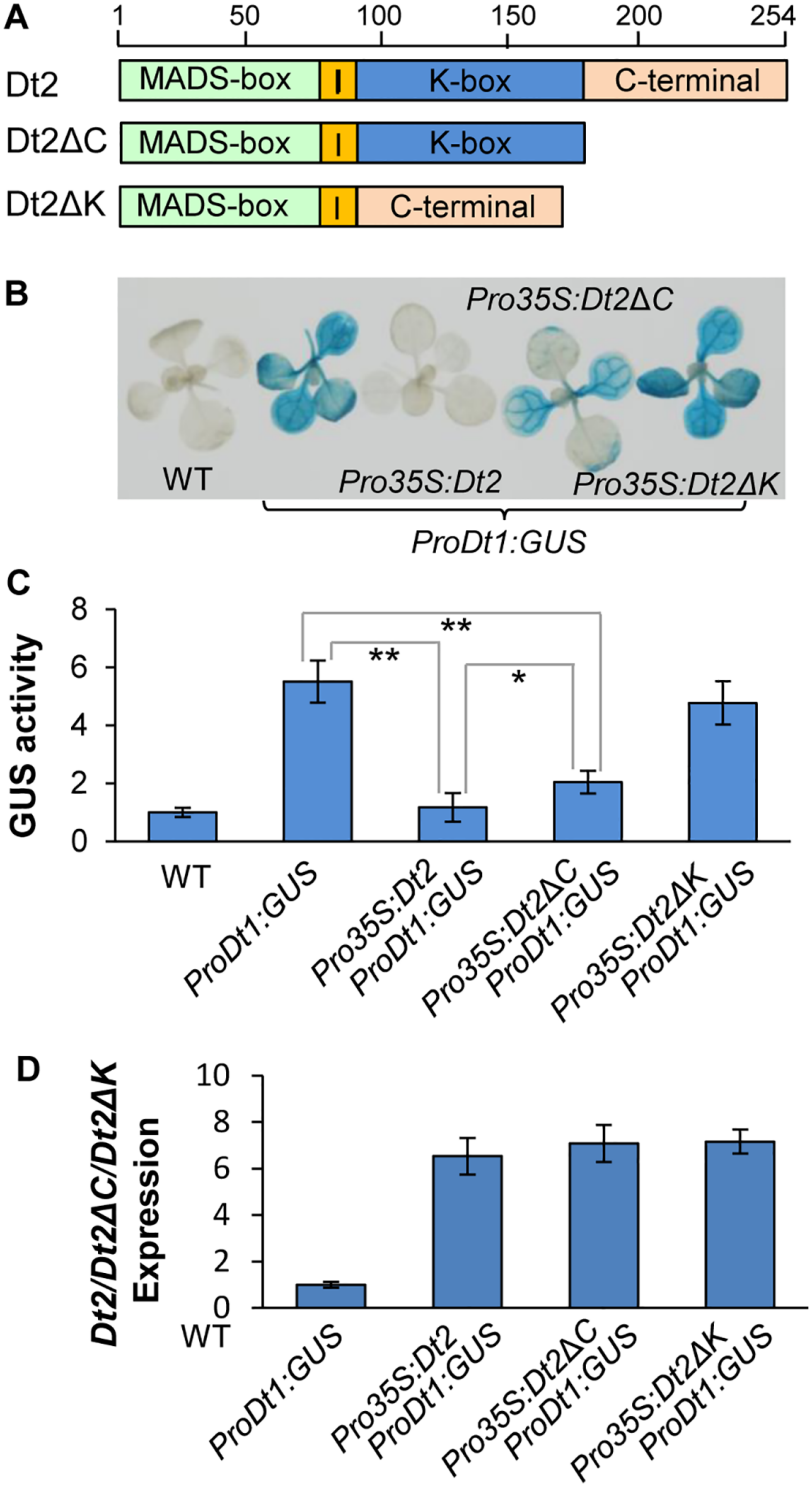
Effects of the K-domain of Dt2 on *Dt1* promoter activity. (A) Diagrammatic Illustration of the conserved motifs in the Dt2 CDS that encode MADS-domain, I-domain, K-domain and C-terminal domain, respectively. (B) GUS staining of the Arabidopsis seedlings with different combination of the transgenes. Proportions of the Dt2 CDS integrated into plasmids for transformation are shown in the (A). (C) Quantitative measurement of GUS activities in the intact seedlings of individual Arabidopsis lines. Doubled-asterisks and asterisks indicate significant differences between compared samples at the levels of p<0.01 and p<0.05 (t-test). (D) Expression levels of the Dt2, Dt2ΔC, Dt2ΔK of under the control of the 35S promoter in the Arabidopsis seedlings and the presence of ProDt1:GUS. The “expression level” of Dt2 in the ProDt1-GUS transgenic line (as a control) was set as 1, and those of Dt2, Dt2ΔC, Dt2ΔK in three corresponding transgenic lines were adjusted accordingly. Values in (D) are shown as mean δ standard errors of the means from three technical replicates.

In addition to the MADS-box domain that binds to the three CArG-boxes in the *Dt1* promoter region, a Keratin (K)-box domain, I domain, and a C-terminal or C domain were predicted in the Dt2 protein based on the homolog searches against the conserved domain database (Fig 3A). Compared with MADS-box domains, K-domains are generally less conserved and often involved in protein-protein interactions to form heterodimers for performing their functions [40–43]. It was reported that the C-domains of some MADS-box factors are also important for translational regulation [13]. To test if the K-domain and C-domian in Dt2 are required for fulfillment of the Dt2 function of repressing *Dt1* expression, we investigated the effects of constitutive expression of the intact Dt2 protein, an incomplete Dt2 protein without the K-domain (Dt2ΔK), and an incomplete Dt2 protein without a C terminal domain (Dt2ΔC), driven by the 35S promoter, respectively (Fig 3A), on the activity of the *Dt1* promoter in Arabidopsis using GUS as a reporter. As shown in Fig 3B and 3C, the activity of the *Dt1* promoter was inhibited under the constitutive expression of *Dt2*, was partially inhibited under the constitutive expression of *Dt2ΔC*, and was not inhibited under the constitutive expression of *Dt2ΔK*. By contrast, the expression levels of *Dt2*, *Dt2ΔC*, *Dt2ΔK* did not show obvious difference in levels of expression under the control of the 35S promoter (Fig 3D). These observations suggest that the K-domain is essential, and perhaps, so are its interacting proteins, for the Dt2 function of repressing *Dt1* expression.

**Fig 4:**
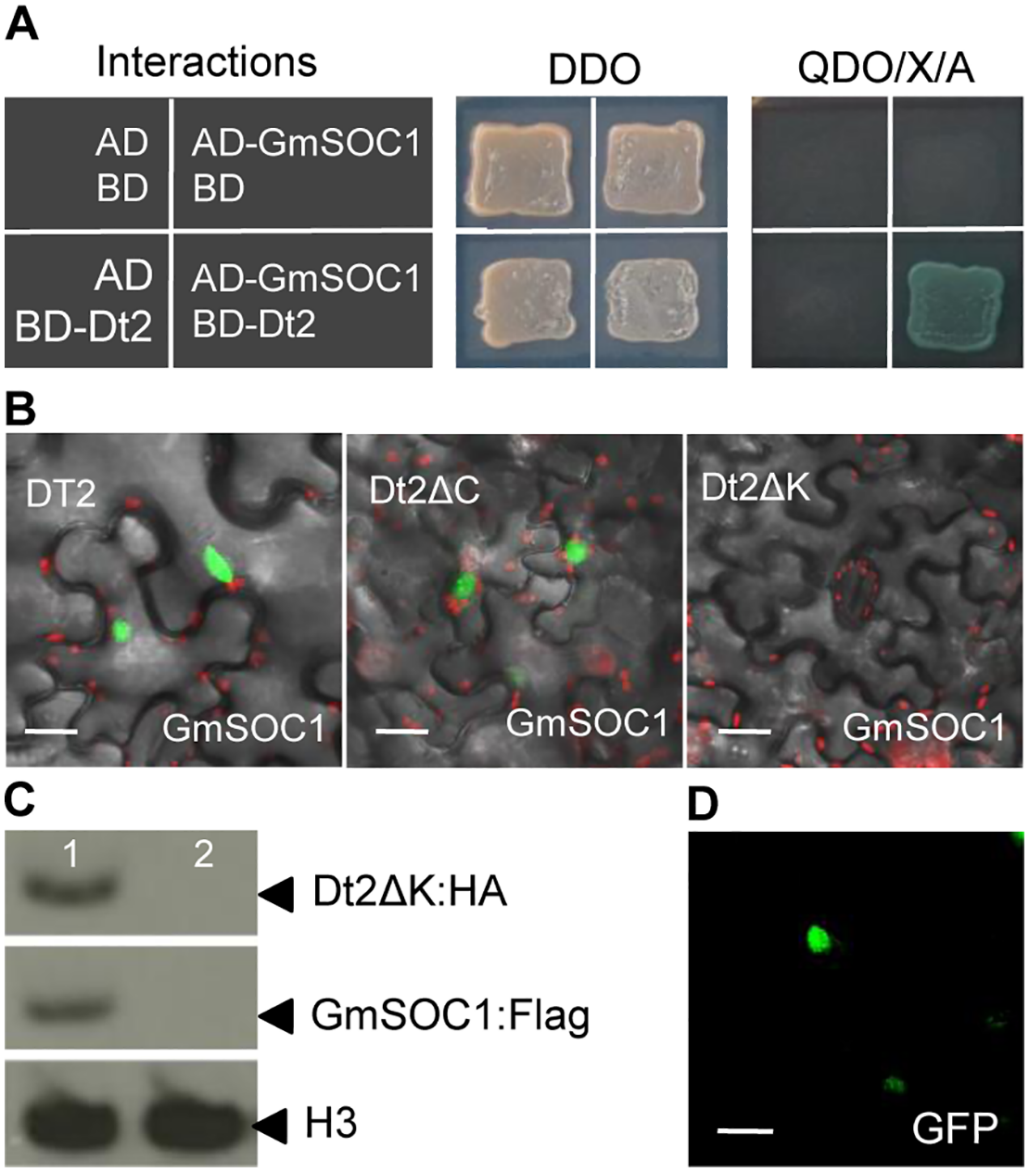
Interaction between Dt2 and GmSOC1 revealed by the Y2H and BiFC assays. (**A**) Dt2 and GmSOC1 interaction validated by Y2H. Double dropout (DDO) medium was used for selection of bait and pray plasmids, while quadruple dropout medium supplemented with X-a-Gal and Aureobasidin A (QDO/X/A) was used to select for the bait and prey plasmids and for confirmation of protein-protein interaction. (**B**) Essential role of the K-domain of Dt2 in interacting with GmSOC1 in tobacco epidermal cells under control of the 35S promoter revealed by BiFC. The portions of the Dt2 protein for Dt2Δc and Dt2Δk, are shown in Fig 3A. (**C**) Expression of the Dt2ΔK:HA fusion protein and the GmSOC1:FLAG fusion protein in the co-infiltrated leaf epidermal cells of 3- to 4-week old tobacco detected by the HA-antibody (anti-HA) and the FLAG antibody (anti-FLAG) through Western blotting, using the Histone H3 protein detected by the H3 antibody (anti-H3) as a control. Sample 1 (left) is co-infiltrated leaf epidermal cells cells with a combination of Dt2ΔK:pEarleyGate201-YN, GmSOC1: pEarleyGate202-YC and p19, and the sample 2 is infiltrated cells with p19 only (**D**) Subcellular localization of the GmSOC1-GFP fusion protein in tobacco epidermal cells under control of the 35S promoter. Scale bars: 50μm.

We then carried out Yeast Two Hybrid (Y2H)-screening of a cDNA library constructed with V2-stage stem tips of soybean using Dt2 as the bait, and identified eight unique cDNA clones each with an insert from a soybean gene (S2 Table), including a putative orthologs of the Arabidopsis *SUPPRESSOR OF OVEREXPRESSION OF CONSTANS 1* (*SOC1*), dubbed *GmSOC1*. *SOC1* in Arabidopsis encodes a MADS-domain factor protein, which integrates multiple flowering signals derived from photoperiod, temperature, hormone, and age-related signals [44, 45]. However, similar to *AP1*, *SOC1* is not expressed in the main shoot of Arabidopsis to maintain the stem’s indeterminate growth [2]. The interaction between Dt2 and GmSOC1 was further validated by Y2H (Fig 4A) and bimolecular florescence complementation (BiFC) using leaf cells of *Nicotiana benthammiana* (Fig 4B). The interaction signals between Dt2ΔC and GmSOC1 were detected by BiFC, whereas no interaction signals between Dt2ΔK and GmSOC1 were detected. Because both the Dt2ΔK and GmSOC1 proteins were expressed at substantially high levels (Fig 4C), the lack of interaction signals between Dt2ΔK and GmSOC1 would be indicative of the lack of interaction between the two proteins. These results are consistent with the ineffectiveness of *Dt2ΔK* expression on suppression of *Dt1* expression, as illustrated in Fig 3, suggesting that Dt2 interacts with GmSOC1 via its K-domain, and that GmSOC1 is important for fulfillment of the Dt2 function. As a MADS-domain factor, GmSOC1, as expected, was localized to the nucleus (Fig 4D).

### GmSOC1 Directly Binds to the *Dt1* Promoter to Repress *Dt1* Expression

**Fig 5:**
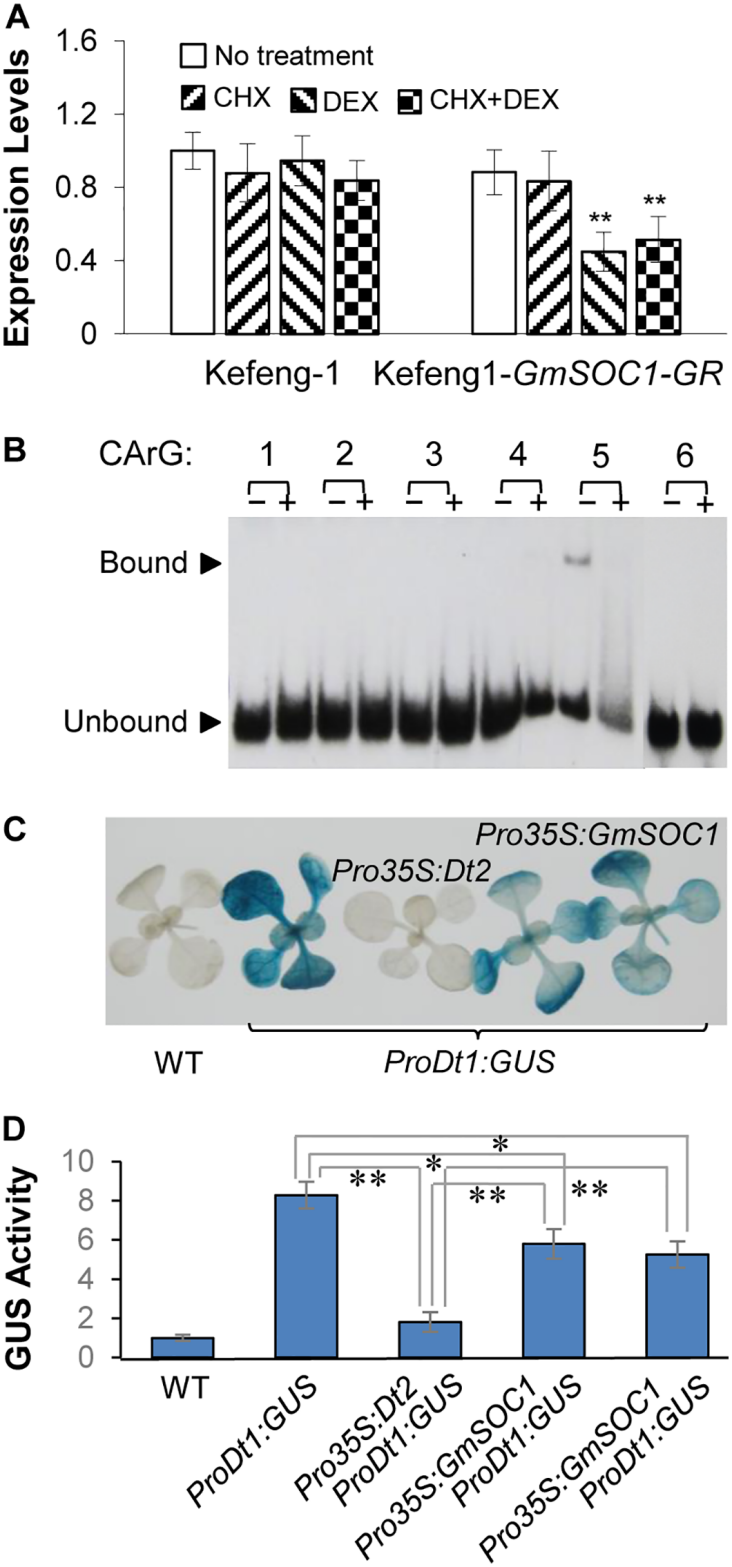
Binding of GmSOC1 to the promoter region of *Dt1* repressing its expression. (**A**) Expression levels of *Dt1* in the *GmSOC1-GR* non-transgenic and transgenic roots of Kefeng 1 under different treatments. Expression level of *Dt1* in the untreated sample of each group was set as 1.0, and those of the other three samples were adjusted accordingly. Values are shown as mean δ standard errors of the means from three biological replicates. Doubled-asterisks indicate significant difference compared with DEX-treated samples (*t*-test, *p*<0.01). (**B**) GmSOC1-binding site in the regulatory region of *Dt1* revealed by EMSA. The “+” and “−” indicate reactions with and without unlabeled competitor probes, respectively. Upper arrow indicated probes bound by the GmSOC1-6×His fusion protein, and the lower arrow indicates probes unbound by the fusion protein. (**C**) GUS staining of the Arabidopsis seedlings with different combination of the transgenes. (**D**) Quantitative measurement of GUS activities in the intact seedlings of individual Arabidopsis lines.

To test whether GmSOC1 interacts with *Dt1*, a GmSOC1-GR fusion protein was created and expressed in the hairy roots of Kefeng 1 directed by the 35S promoter to evaluate the effect of GmSOC1 activation in the GmSOC1-GR fusion protein on the expression of *Dt1*. The levels of *Dt1* mRNA was reduced significantly in the presence DEX, and in the presence of DEX and CHX, but was not changed significantly in the presence of CHX, compared with the transgenic roots without any treatment (Fig 5A), suggesting that GmSOC1 was involved in repression of *Dt1* transcription by direct binding to the promoter region of *Dt1*. Nevertheless, the effect of constructive expression of *GmSOC1* on repression of the *Dt1* promoter activity is not as strong as the effect of constructive expression of *Dt2* on repression of the *Dt1* promoter activity (Fig 5C and 5D). This is also consistent with the observed effects of GR-fusion proteins on *Dt1* expression (Figs 2A and 5A).

A GmSOC1-6×His fusion protein was isolated from the *Escherichia coli* strain BL21 and used to examine whether the MADS-domain of GmSOC1 can bind to any of the CArG-boxes in the promoter region of *Dt1* by EMSA. As shown in Fig 5B, only binding of GmSOC1 with the 5^th^ CArG box in the *Dt1* promoter region was detected.

### Spatiotemporal Co-expression of *Dt2* and *GmSOC1* Determines Semi-determinacy

**Fig 6:**
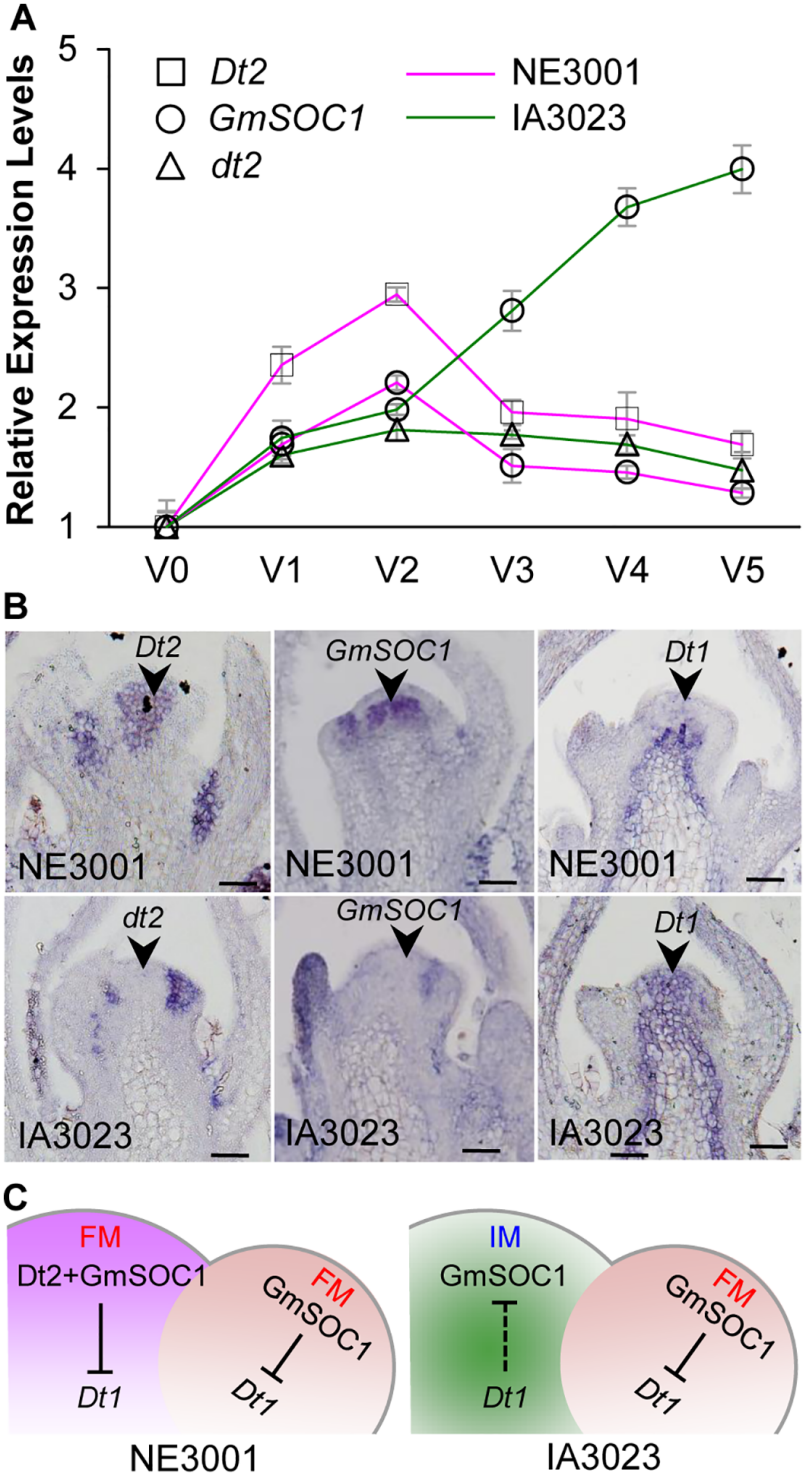
Expression patterns of *Dt2/dt2*, *GmSOC1*, and *Dt1* and their interactions. (**A**) Expression levels of *Dt2/dt2* and *GmSOC1* in stem tips of NE3001 and IA3023 from the V0 to V5 stages. Values are shown as mean δ standard errors of the means from three biological replicates. Expression level of each gene/allele at the V0 stage was set as 1, and those at other stages were adjusted accordingly. (**B**) Spatial expression of *Dt2/dt2*, *GmSOC1*, and *Dt1* in the stem tips of NE3001 and IA3023 at the V2 stage detected by RNA *in situ* hybridization. Arrows point to SAMs. Scale bars: 50μm. (**C**) Diagrammatic illustration of spatial expression of *Dt2/dt2*, *GmSOC1*, and *Dt1* in stem tips of NE3001 and IA3023 at the V2 stage and distinct mechanisms underlying floral initiation between SAMs and lateral meristems. Solid lines indicate validated interactions, while the dotted line indicates a deduced interaction.

Expression analysis by qRT-PCR using the stem tips from NE3001 and IA3023 revealed consistent expression patterns between *GmSOC1* and *Dt2* in NE3001 from the V0 stage through the V5 stage (when the fourth trifoliate leaflets are fully expanded but before the fifth trifoliate leaflets are still unrolled) (Fig 6A). It is particularly noticeable that both *GmSOC1* and *Dt2* were expressed at the highest levels in NE3001 at the V2 stage. By contrast, *GmSOC1* in IA3023 showed an expression pattern distinct from *dt2*. In particular, the expression levels of *GmSOC1* in IA3023 continued to be elevated after the V2 stage through the V5 stage, suggesting that *GmSOC1* may have different regulatory roles between the *Dt2* and *dt2* backgrounds.

Given that *Dt2* is primarily expressed in the stem tips, we thus performed *in situ* hybridization to localize the transcripts of *Dt2/dt2*, *GmSOC1*, and *Dt1* alleles in specific sections within the V2-stage stem tips in semi-determinate NE3001 and indeterminate IA3023 (Fig 6B). It was found that the *Dt2* transcripts were concentrated in the central zone of the SAMs at the V2 stage in NE3001, where the *Dt1* expression was not detected. By contrast, the *dt2* transcripts were not detected in the central zone of SAMs in IA3023, where abundant *Dt1* transcripts were observed. In the *Dt2* (i.e., NE3001) background, the *GmSOC1* transcripts were detected in SAMs, whereas no expression of *GmSOC1* was detected in SAMs in the *dt2* (i.e., IA3023) background. The spatiotemporally specific co-expression of *Dt2* and *GmSOC1* in NE3001 and absence of *dt2* and *GmSOC1* transcripts in SAMs of IA3023 suggest that the observed expression of *GmSOC1* in SAMs and thus its novel function are *Dt2*-depedent, and that both *Dt2* and *GmSOC1* are involved in transcriptional repression of *Dt1*, responsible for the formation of semi-determinacy.

It is also noticeable that *dt2* was expressed in lateral meristems in IA3023, where the transcripts of *Dt1* was not detected, and that *GmSOC1* was detected in lateral meristems in both NE3001 and IA3023, where no or minimal expression of *Dt1* was observed (Fig 6B), suggesting that the lateral meristems at the stem tips of the V2 stage may be in the state of transition from IMs to FMs in both NE3001 and IA3023, and that both *dt2* and *GmSOC1* may be involved in floral induction in the lateral meristems of both NE3001 and IA3023, most likely, by suppressing *Dt1* transcription, as *Dt2* and *GmSOC1* do in the SAMs of NE3001 (Fig 6C).

### Heterologous Expression of *Dt1* and *Dt2* Creates Semi-determinacy in Arabidopsis

Our previous study demonstrated that the *TFL1/tfl1* promoter was able to drive the expression of *Dt1* in the Arabidopsis *tfl1* mutant to convert the mutant phenotypes (determinate stem and early flowering) to the wild-type phenotypes (indeterminate stem and late flowering) [17]. Also, since the activity of the *Dt1* promoter could be detected by the GUS gene in Arabidopsis (Fig 3B), we were thus curious about whether the expression of *Dt1* driven by its own promoter in Arabidopsis *tfl1* mutants could recover the wild-type phenotype, and if so, whether ectopic expression of *Dt2* alone or in combination with *Dt1* in the *tfl1* mutant could produce semi-determinate stem growth habit that has not been observed in Arabidopsis.

**Fig 7:**
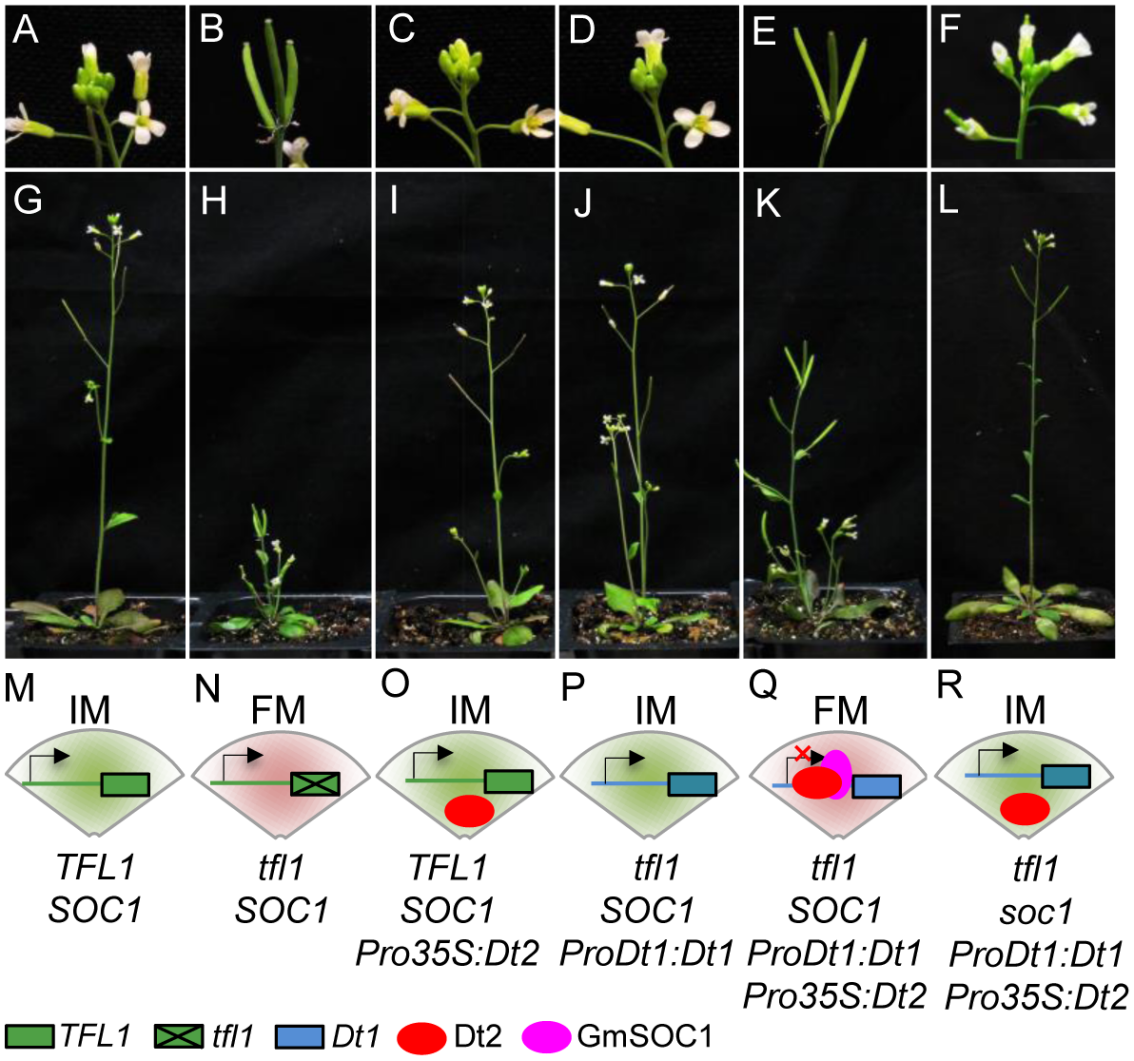
Heterologous expression of *Dt2* and/*or Dt1* in *tfl1* and wild type Arabidopsis. The racemes (**A-F**) of the main stems of their respective plants (**G-L**), and the genotypes and interactions underlying the identity of SAMs (**M-R**) are shown as photographs and schematic diagrams, respectively. Arrows indicates transcriptional orientation and the red “×” indicates that the *Dt1* expression is repressed.

To address these questions, we created a *ProDt1*:*Dt1* construct comprised of the promoter of *Dt1* and its rest genomic sequence, and introduced it to an Arabidopsis *tfl1* mutant line to produce *ProDt1*:*Dt1* transgenic lines. We also developed *Pro35S*:*Dt2* transgenic lines with the wild-type (*TFL1*) Arabidopsis. These two transgenic lines were crossed to generate progeny lines containing both *ProDt1*:*Dt1* and *Pro35S*:*Dt2* in the *tfl1* background. We found that the *ProDt1*:*Dt1* transgenic line with the *tfl1* background recovered the wild-phenotypes that are typically shown by the wild-type Arabidopsis (Fig 7D and 7J), suggesting that the *Dt1* promoter functions as the *TFL1/tfl1* promoter in driving *Dt1* expression to fulfill the *TFL1* function. The ectopic expression of *Dt2* in the wild-type genetic background did not affect the indeterminate stem growth determined by *TFL1*. By contrast, the ectopic expression of *Dt2* expression in the *ProDt1*:*Dt1* transgenic line with the *tfl1* background exhibited semi-determinate stem growth habit similar to shown by semi-determinate soybean (Fig 7E and 7K), indicating that the *TFL1* promoter activity was not repressed by Dt2.

The spatiotemporal co-expression of Dt2 and GmSOC1 and their co-binding to the Dt1 promoter suggests the essential role of GmSOC1 in the formation of semi-determinacy. To further test this hypothesis, we crossed the semi-determinate Arabidopsis transgenic line (Fig 7E and 7K) with the Arabidopsis *soc1* mutant [44,45], and obtained F_2_ plants containing both *ProDt1*:*Dt1* and *Pro35S*:*Dt2* in the *tfl1 and soc1* background. As exemplified in Fig 7F and 7L, the plants with such a combination of genes showed indeterminate stem growth, indicating that *SOC1* is indeed essential to repress *Dt1* transcription in the *Dt2* background in SAMs.

## Discussion

We present a novel mechanism underlying plant stem terminal flowering and semi-determinate growth habit, a key adaptation and agronomic trait that was formed post-domestication of soybean and was artificially selected for soybean production by breeding [23]. We demonstrate that the spatiotemporal expression of a recent gain-of function mutation allele *Dt2* in apical meristems of main stems triggers co-expression of the putative soybean floral integrator gene *GmSOC1*, and the proteins encoded by these two genes directly interact to form co-repressors to directly target and repress *Dt1* transcription, resulting in the formation of apical floral meristems that is essential for semi-determinate stem growth habit. In Arabidopsis and all other plant species that have been investigated to date, terminal flowering is achieved by the null mutations of *TFL1* or its functional equivalents [9, 10, 17–22, 30]. Thus, our findings represent a unique mechanism reshaped by artificial selection. Similar to the “green revolution” semi-dwarf trait in cereals [46], semi-determinacy makes soybean plants more lodging-resistant and thus is desirable for production in high-yield lodging-prone environments. In addition, we demonstrate potential application of this mechanism for modification of stem growth habit in other species.

### *Dt1* is the Functional Equivalent of the Arabidopsis *TFL1*

As reflected by the timing and spatial patterns of it’s expression, *Dt1* in indeterminate soybean appears to function in a way similar to what *TFL1* does in the wild type Arabidopsis to repress stem terminal flowering. In Arabidopsis, *TFL1* starts to be expressed weakly in the center of the SAMs during the vegetative phase and its expression level is up-regulated at the stage when the SAMs make cauline leaves that bear shoot meristems in their axils [47, 48]. The expression of *TFL1* remains in SAMs of the main shoot afterwards to repress the expression of the floral identity genes such as *AP1*, *LEAFY* and thus sustain the indeterminate growth until the plant cease to grow [35]. In emerging floral meristems on the flanks, *AP1* and *LFY* were found to repress *TFL1* by direct binding to its 3’ regulatory region [15, 16]. A more recent study demonstrated that AP1 recruits SEP4, SOC1, AGL24, and SVP to form a regulatory complex that represses the expression of *TFL1* to initiate lateral flowering [49]. In soybean, the highest level of *Dt1* in main stem tips was detected at the V0 stage, which seems to be equivalent to the Arabidopsis stage when *TFL1* is up-regulated. Because the functional equivalents of the Arabidopsis floral identity genes such as *AP1* and *LEAFY* in soybean have not been identified, it remains unclear when the floral meristems at the flanks are exactly initiated in soybean. Nevertheless, *Dt1* expression was detected in the center zone of SAMs of IA3023, but not detected in the lateral meristems in either NE3001 or IA3023, suggesting that floral induction may have occurred in the lateral meristems in both varieties.

### *Dt2* Is Not the Functional Equivalent of the Arabidopsis *AP1*

Several lines of evidence indicate that *Dt2* is not the functional equivalent of the Arabidopsis of *AP1*, although the functional orthologs *Dt1* and *TFL1* are their respective direct targets. Firstly, *Dt2* is a rare gain-of-function allele that is present only in semi-determinate soybean, while *AP1* is a floral identity gene in the wild-type Arabidopsis; Secondly, *Dt2* is not one of the four soybean duplicates orthologous to the Arabidopsis *AP1* [32]. Instead, *Dt2* appears to be an ancestral copy of MADS-box factor gene proceeding the divergence of *AP1* from *FUL* that had occurred before the split of Arabidopsis from soybean; Thirdly, *Dt2* is expressed in the central zone of SAMs to repress the expression of *Dt1* (Fig 6B), whereas *AP1* is not expressed in SAMs of the main shoots of Arabidopsis [35]. Fourthly, Dt2 binds to the promoter region of *Dt1* (Fig 2E, 2F and 2G), whereas AP1 binds to the 3’ regulatory region of *TFL1* to achieve suppression of the transcription of the two target genes [13]. Nevertheless, *Dt2* appears to be responsible for initiation of floral meristems in SAMs similar to that was achieved by *AP1* in the lateral meristems in Arabidopsis.

### *GmSOC1* Appears to Be The Functional Equivalent of the Arabidopsis *SOC1*

The transcripts of *GmSOC1* were detected not only in the SAMs of NE3001, but also the lateral meristems of both NE3001 and IA3023 by *in situ* hybridization (Fig 6B). Further, the expression level of *GmSOC1* in the main stems with both apical and lateral meristems continues to increase in IA3023 but that starts to decrease in NE3001 after the V2 stage (Fig 6A). Because the main stems of IA3023 continue vegetative growth at their apical meristems and floral induction at their flanks until all meristems are consumed and the plants get matured, whereas the main stems of NE3001 appear to have undergone the transition from IMs to FMs at the V2 stage (Fig 6B), the elevated expression levels of GmSOC1 in IA3023 after the V2 stage would be considered as additional evidence in support of the role of *GmSOC1* as a floral identity gene in soybean. In Arabidopsis, *TFL1* expression is repressed by SOC1 in an AP1-dependent manner [49]. Contrastingly, the repression of *Dt1* by GmSOC1 appears to be Dt2-dependent. Such a similarity reflects not only the functional conservation between GmSOC1 and SOC1 as repressors of *Dt1/TFL1*, but also the way in which they function.

Although the *dt2* transcripts were also detected in the lateral meristems of IA3023 (Fig 6B), the expression level of *dt2* was declined after the V2 stage in IA3023, suggesting that, unlike *GmSOC1*, *dt2* may not be essential for floral induction in the lateral meristems. If *GmSOC1*, indeed, is the functional equivalent of the Arabidopsis *SOC1*, as directly indicated by the recovered indeterminacy by *Dt1*, with the constitutive expression of *Dt2*, in the tfl1 and *soc1* double mutants of Arabidopsis (Fig 7F and 7L), the expression of *GmSOC1* would be essential for initiating terminal flowering through suppressing *Dt1* expression and perhaps through activating the expression of other flowering identity genes in soybean such as functional equivalents of the Arabidopsis *LEAFY* and *FUL* in SAMs.

### Novel Functions of *Dt2* and *GmSOC1* Are Attributed to Their Spatiotemporal Expression

Several observations obtained in this study, such as the spatially specific and co-expression pattern of *GmSOC1* and *Dt2* (Fig 6), their direct interaction (Fig 4A and 4B), the lack of Dt2Δk for interacting with GmSOC1 and repressing *Dt1* expression (Fig 3B and 3C), and the repressive effect of GmSOC1 on *Dt1* expression (Fig 5C and 5D), suggest that *GmSOC1* plays an essential role in forming the semi-determinate stem growth habit, and this role was likely fulfilled by its dimerization with Dt2. As the Arabidopsis *SOC1* is not expressed in the shoot meristems, such a pattern of *GmSOC1* expression would indicate its novel function, which was specifically triggered by *Dt2*. As similarly observed in Arabidopsis, rice *SOC1*, *AGL24*, *SVP*, and *SEP4* orthologs regulate panicle branching through suppressing the *TFL1* orthologs in rice, indicating the genetic pathways underlying inflorescence architecture are highly conserved between monocot and dicot species [49]. Intriguingly enough, such an inter-specifically conserved pathway is not conserved between the apical and lateral meristems in initiating flowering in semi-determinate soybean due to the spatiotemporal expression of *Dt2* (Fig 6). It is possible that the activation of *GmSOC1* was initiated through suppression of *Dt1* by Dt2. Alternatively, *GmSOC1* could be directly activated by Dt2. It would be interesting to further investigate how such specific expression of *Dt2* was achieved simply through the gain-of-function mutation(s) that occurred outside of its CDS [32], and how the spatiotemporal expression of *GmSOC1* was triggered by the *Dt2* mutation, and to what extent the regulatory networks underlying soybean stem growth habit was reshaped by the *Dt2* mutation.

### Heterologous Expression of *Dt2* and *Dt1* in Arabidopsis: Conservation, Divergence, and Application

The formation of the semi-determinate Arabidopsis by heterologous expression of *Dt2* and *Dt1* in the *tfl1* mutants is an applausive observation (Fig 7), which suggests that all the functions of the soybean genes involved in the regulatory complex suppressing *Dt1* expression can be fully provided by the Arabidopsis genes. By contrast, the overexpression of *Dt2* in wild Arabidopsis did not result in any stem architectural changes, suggesting that Dt2 did not interact with *TFL1*. This may be explained by the absence of any CArG-boxes in the promoter region of *TFL1* as potential target sites of Dt2. Therefore, the heterologous expression experiment demonstrated both conservation and divergence of regulatory sequences between *TFL1* and *Dt1* for precise control of their switch-on and switch-off. It would be important to further identify floral identity genes in FMs developed from the apical IMs and secondary IMs, respectively, towards a more in-depth understanding of the spatiotemporal specificity and commonality of floral regulation that determine that plant’s inflorescence architecture.

Given such a long period of divergence of soybean and Arabidopsis from a common ancestor, the formation of semi-determinacy by *Dt2* and *Dt1* in Arabidopsis would suggest a feasibility and potential application of this novel regulatory mechanism for modification of stem growth habit in many other plants, particularly, the legume crops, towards optimizing plant architecture for enhanced yield potential and adaptability.

## Materials and Methods

### Plant Materials

Semi-determinate elite soybean line NE3001 (*Dt2Dt2;Dt1Dt1*), indeterminate soybean elite lines IA3023 and Thorne (*dt2dt2;Dt1Dt1*), and a Dt2 over-expression transgenic line (#2) in the Thorne genetic background were previously described [32]. An indeterminate elite line Kefeng 1 (*dt2dt2;Dt1Dt1*), used for hairy root transformation, was obtained from the USDA Soybean Germplasm Collection. The *tfl1* mutant (*tfl1-1*) was obtained from The Arabidopsis Information Resource (TAIR) Arabidopsis Stock Centers. The Arabidopsis seeds were surface-sterilized with 10% bleach plus 0.01% Triton X-100 for 12 min, followed by washing five times with sterile water. The sterilized seeds were stratified at 4°C for 2 days and transferred to culture media or soil for further growth at 22°C under the condition of 16 h of 120 μE·m^−2^·s^−1^ light and 8 h of dark.

### DNA and RNA isolation, PCR, and Sequencing

Genomic DNA isolation, PCR primer design, PCR amplification with genomic DNA, PCR product purification, RNA isolation, cDNA synthesis by reverse transcription-PCR (RT-PCR), quantitative real-time-PCR (qRT-PCR), and sequencing of DNA and cDNA fragments were performed using protocols previously described [17, 32]. In the qRT-PCR experiments, three biological replicates were analyzed to quantify the levels of gene expression in NE3001, IA3023, Kefeng 1 and three technical replicates were performed to measure the levels of gene expression in Thorne and the Thorne transgenic line, and the soybean ATP binding cassette transporter gene *Cons4* [39] was used as the internal control, and we normalized the relative expression levels of the genes/alleles *Dt2*, *dt2*, *Dt1*, *GmSOC1* in each experiment by setting the lowest expression level as 1.0. Primers used for PCR, RT-PCR, qRT-PCR and sequencing are listed in S3 Table.

### Plasmid Construction and Plant Transformation

The full-length or portions of the CDSs of *Dt2*, and *GmSOC1* were amplified by RT-PCR using KOD hot start DNA polymerase (Novagen catalog no. 71087), and the *Dt2*Δ*K* fragments were obtained by overlapping PCR with two overlapped CDS fragments as templates in a same reaction. The CDS and fused CDS fragment were then inserted to *pCR8/GW/TOPO* vector (Invitrogen, catalog no. K2500-20) and verified by sequencing. Subsequently, the verified inserts were cloned into the binary vector *pGWB17* [50] to obtain the three constructs *Pro35S*:*Dt2*, *Pro35S*:*Dt2ΔC*, *Pro35S*:*Dt2ΔK*, and *Pro35S*:*GmSOC1*, and the verified inserts were cloned into *pBI-ΔGR-GW* [51] to generate the *Pro35S*:*Dt2-GR* and *Pro35S*:*GmSOC1-GR* constructs.

The ~2.4-kb upstream sequence from the start codon (dabbed the promoter region or *pProDt1*), the truncated promoter without the cluster of the five putative CArG-boxes (dabbled *ProDt1Δ*), the CDS, and the ~1.1kb downstream sequence from the stop codon (dabbed terminator region) of *Dt1* were amplified from the indeterminate soybean cultivar Williams 82. The obtained PCR fragments were cloned into the *pGEM-T* Easy Vector (Promaga, catalog no. A1360) and then sequenced. The verified clones with the promoter region, the CDS, and the terminator region, were digested by *Pst*I and *Sal*I, *Sal*I and *Xba*I, and *Xba*I and *BamH*I, respectively, and then integrated into *pPZP212* [32]. The construct of *ProDt1Δ*:*LUC* was made by integrating *pProDt1 into pGWB435* [50]. The *Dt1* promoter sequences with point mutations within each of the three CArG boxes were created by using QuikChange II Site-Directed Mutagenesis Kit (Agilent Technologies, Catalog #200523) with specifically designed primers (S3 Table). These constructs were introduced into Agrobacterium tumefaciens strain *GV3101* or *Agrobaterium rhizogenes* strain K599. The Arabidopsis transgenic lines each from a single construct were obtained by *A*. *tumefaciens*-mediated transformation, and the transgenic lines with genes from two distinct constructs were generated by crossing two transgenic lines with respective transgenes and subsequent screening of the progeny lines. The soybean transgenic hairy roots were produced by *A*. *rhizogenes*-mediated transformation following a protocol previously described by Kereszt et al. [40]. The seedlings with transgenic roots were sprayed a solution of 0.03mM DEX with 0.005% Silwet L-77, a solution of 1.8mM CHX with 0.005% Silwet L-77 or a solution of 0.03mM DEX and 1.8mM CHX with 0.005% Silwet L, and levels of gene expression was measured four hours after each treatment.

### Antibody Synthesis and ChIP Assays

Thermo Scientific Antigen Profiler, a bioinformatics protein sequence analysis tool and custom peptide design algorithm, was provided by Pierce Biotechnology, Inc. and employed to design a unique 19 amino-acid peptide from Dt2, which was then used to raise a Dt2-specific antibody from a rabbit (dabbed anti-Dt2, Pierce Biotechnology, Inc.). The specificity of the anti-Dt2 was tested by Western blot using total proteins isolated from the stem tips of NE3001 at the V2 stage.

ChIP assays with anti-Dt2 were performed following the protocols described previously [52, 53], with minor modification. Stem tips of NE3001 collected at the V2 stage were immersed in 1×Phosphate Buffered Saline (PBS) buffer containing 1% formaldehyde (Macron, catalog no. K15754) for cross-linking. Enrichment of the precipitated DNA by anti-Dt2 relative to DNA recovered from the control treatment without anti-Dt2 was measured by qRT-PCR with three biological replicates. The primers used in ChIP-PCR are listed in S3 Table.

### EMSA Assays

The CDSs of *Dt2* and *GmSOC1* were cloned into the expression vector *pET-DEST42* containing a 6×His tag (ThermoFisher Scientific, catalog no. 12276–010), separately, to generate the *Dt2-6×His* and *GmSOC1-6×His* constructs. The two constructs were transformed into the *Escherichia coli* strain BL21 and *Rosetta* (*DE3*), respectively, The Dt2-6×His and GmSOC1-6×His fusion proteins were then induced in the transformed cells by growing at 37°C for 5h in the 2×YT medium with 1 mM isopropyl β-D-1- thiogalactopyranoside and then extracted and purified with Ni-NTA Agarose (Qiagen, catalog 30210). EMSAs were performed using digoxigenin-labeled probes and the DIG Gel Shift Kit (Roche, 3353591910) following the manufacturer’s instructions.

### GUS Staining and Activity Measurement

Ten transgenic plants for each construct were mixed for protein extraction and histochemical staining. The Gus activities were measured in a method described earlier [54].

### Y2H cDNA Library Construction and Screening and Y2H Validation

The stem tips from Williams 82 at the V2 stage were used to isolate total RNA, which was used to synthesize cDNA by reverse transcription. The pool of cDNA fragments were cloned into *pGADT7* (Clontech, catalog no. 630442) and then transformed into the yeast strain Y187 (Clontech, catalog no. 630457), following the manufacturer's instructions The CDS of *Dt2* was inserted into vector *pGBKT7 as* a bait and introduced into the yeast strain Y2H Gold (Clontech, catalog no. 630498). Mating between the Dt2 strain and the cDNA library were screened on the quadruple dropout medium (QDO) with SD/–Ade/–His/–Leu/–Trp (Clontech, catalog no. 630322). The positive cDNA clones were sequenced and the interaction between Dt2 and one of the positive clones, which contains a fragment from the CDS of GmSOC1, was further validated by co-transformation of *pGBKT7* with the *Dt2* CDS and *pGADT7* (Clontech, catalog no. 630442) with the *GmSOC* CDS into Y2HGold and grown on the selection medium QDO supplemented with X-a-Gal (Clontech, catalog no. 630462) and Aureobasidin A (Clontech catalog no. 630466) following the manufacturer's instructions.

### BiFC Assays

For BiFC assays, the *Dt2*, *Dt2ΔC*, and *Dt2ΔK* were cloned into the *pEarleyGate201-YN* vector [55] and the *GmSOC1* was cloned into the *pEarleyGate202-YC* vector [55]. These constructs were introduced into the *A*. *tumefaciens* strain GV3101, together with the p19 strain, and the strains carrying *GmSOC1* and *Dt2*, *Dt2ΔC*, or *Dt2ΔK* were co-infiltrated into leaf epidermal cells of 3- to 4-week old tobacco (*Nicotiana benthamiana*), following a protocol described previously [56]. The transformed cells were observed and photographed using a confocal scanning microscope (Nikon 90i) 24 h after infiltration.

#### Protein extraction and western blot analyses

For protein extraction, leaf discs were harvested, frozen in liquid nitrogen and stored at 80°C. Denatured protein extracts were obtained by incubating homogenized plant material in SDS sample buffer [62.5 mM 2-amino-2-(hydroxymethyl)-1,3-propanediol (TRIS), pH 6.8, 2% SDS, 10% glycerol, 5% mercaptoethanol)] for 5 min at 95°C, centrifuged (20 min, 20 000 g, 4°C) and the supernatant was stored at 20°C. Immunodetection was performed using monoclonal HA (C29F4, Cell Signaling), FLAG (F7425, Sigma) and H3 (Histone 3) (9715, Cell Signaling), Anti-rabbit IgG (7074, Cell Signaling; 1:10,000) conjugated with alkaline phosphatase was used as the secondary antibody with an enhanced chemiluminescence protein gel blot detection system (Amersham, Sweden).

### Subcellular Localization

For subcellular localization, the CDS of *GmSOC1* was cloned into the plasmid *pGWB405* to form a fusion protein of GmSOC1 and a green fluorescent protein (GFP) under the control of 35S promoter, which are provided by the plasmid. The construct was introduced into leaf epidermal cells of 3- to 4-week old tobacco by *A*. *tumefaciens* infiltration. The transformed cells were observed and photographed using a confocal scanning microscope (Nikon 90i) 24 h after infiltration.

### RNA *In Situ* Hybridization

RNA *in situ* hybridization was performed according to a previously described protocol [57]. A 120-bp fragment specific to the *Dt2/dt2* cDNA, a 123-bp fragment specific to the *Dt1* cDNA, and a 188-bp fragment specific to the *GmSOC1* cDNA were amplified with respective primer sets (S3 Table), and then integrated into the *pGEM-T* Easy vector, respectively. Digoxigenin-labeled sense and anti-sense probes were obtained from EcoR-digested linear *pGEM-T* Easy Vectors with 120-bp, 123-bp, or 188-bp inserts by in vitro transcription with SP6 or T7 RNA polymerase (Roche, catalog no. 11175025910) according to the manufacturer’s protocol. Hybridization signals were detected and photographed using a confocal scanning microscope (Nikon A1R).

## Supporting Information

S1 Fig Two elite soybean cultivars with distinct stem growth habit. Indeterminate cultivar IA3023 (left), and semi-determinate cultivar NE3001 (right).
(PPTX)

S2 Fig Peptides in the MADS-domain of Dt2 used to raise the Dt2 antibody. Alignments of predicted amino acid sequences encoded by Dt2 and other three genes showing highest levels of sequence similarity. Peptides used to raise the Dt2 antibody is framed.
(PPTX)

S1 Table A regulatory mechanism modulating semi-determinate stem growth habit innovated by artificial selection in soybean. (DOCX)

S2 Table Nine genes identified by Y2H screening as candidates encoding proteins that interact with Dt2. (DOCX)

S3 Table Primers used for PCR, RT-PCR, qRT-PCR, ChIP-PCR. (DOCX)
