# Supplementary material for: Innovation of a Regulatory Mechanism Modulating Semi-determinate Stem Growth through Artificial Selection in Soybean
Source: PLoS Genet. 2016 Jan 25;12(1):e1005818. doi: 10.1371/journal.pgen.1005818 (PMC4726468; doi:10.1371/journal.pgen.1005818)
Supplement: S1 Fig — Indeterminate cultivar IA3023 (left), and semi-determinate cultivar NE3001 (right). (PPTX) [file pgen.1005818.s001.pptx]

## Slide 1
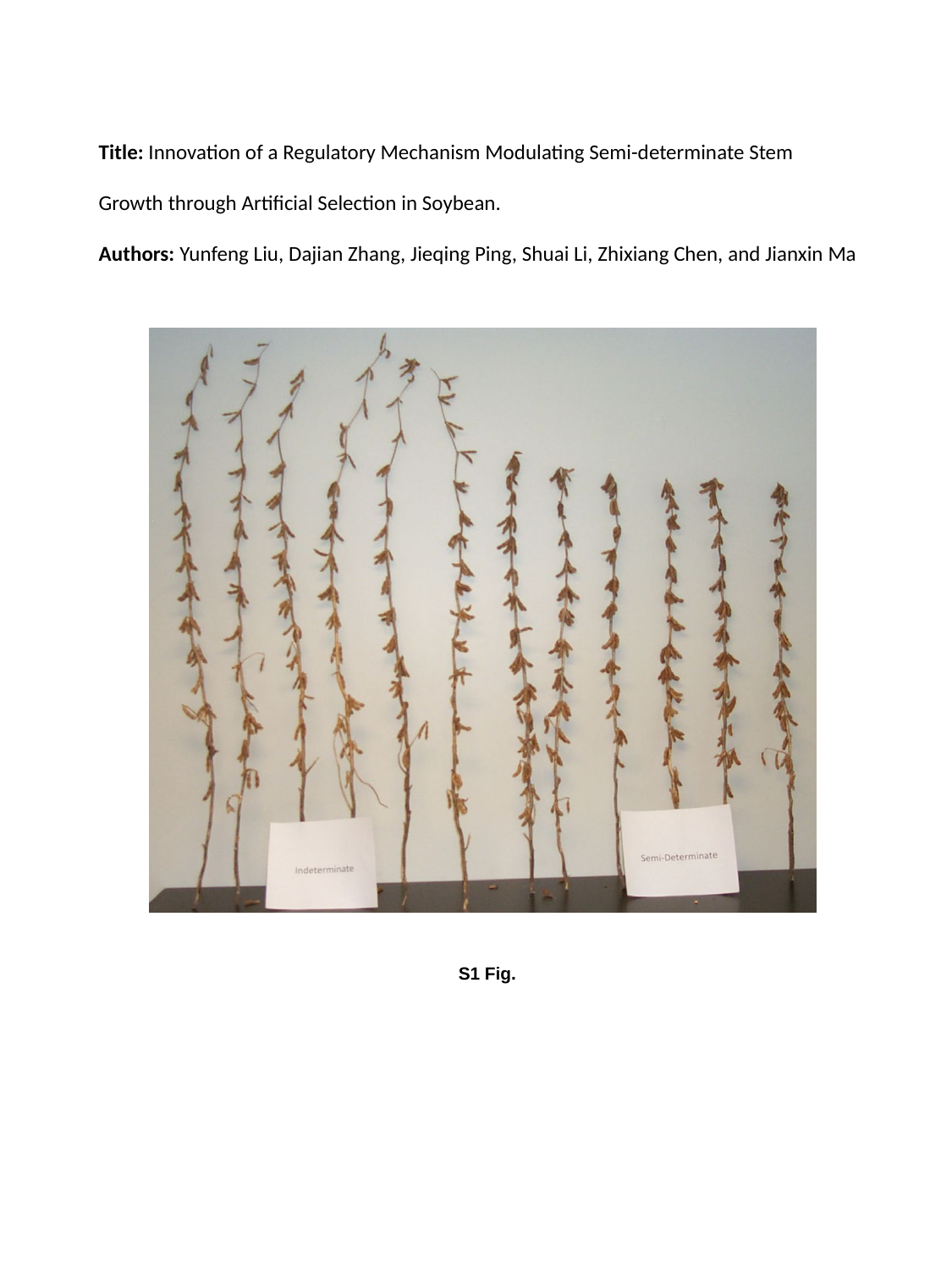

Title: Innovation of a Regulatory Mechanism Modulating Semi-determinate Stem Growth through Artificial Selection in Soybean.
Authors: Yunfeng Liu, Dajian Zhang, Jieqing Ping, Shuai Li, Zhixiang Chen, and Jianxin Ma
S1 Fig.
