# Supplementary material for: Innovation of a Regulatory Mechanism Modulating Semi-determinate Stem Growth through Artificial Selection in Soybean
Source: PLoS Genet. 2016 Jan 25;12(1):e1005818. doi: 10.1371/journal.pgen.1005818 (PMC4726468; doi:10.1371/journal.pgen.1005818)
Supplement: S2 Fig — Alignments of predicted amino acid sequences encoded by Dt2 and other three genes showing highest levels of sequence similarity. Peptides used to raise the Dt2 antibody is framed. (PPTX) [file pgen.1005818.s002.pptx]

## Slide 1
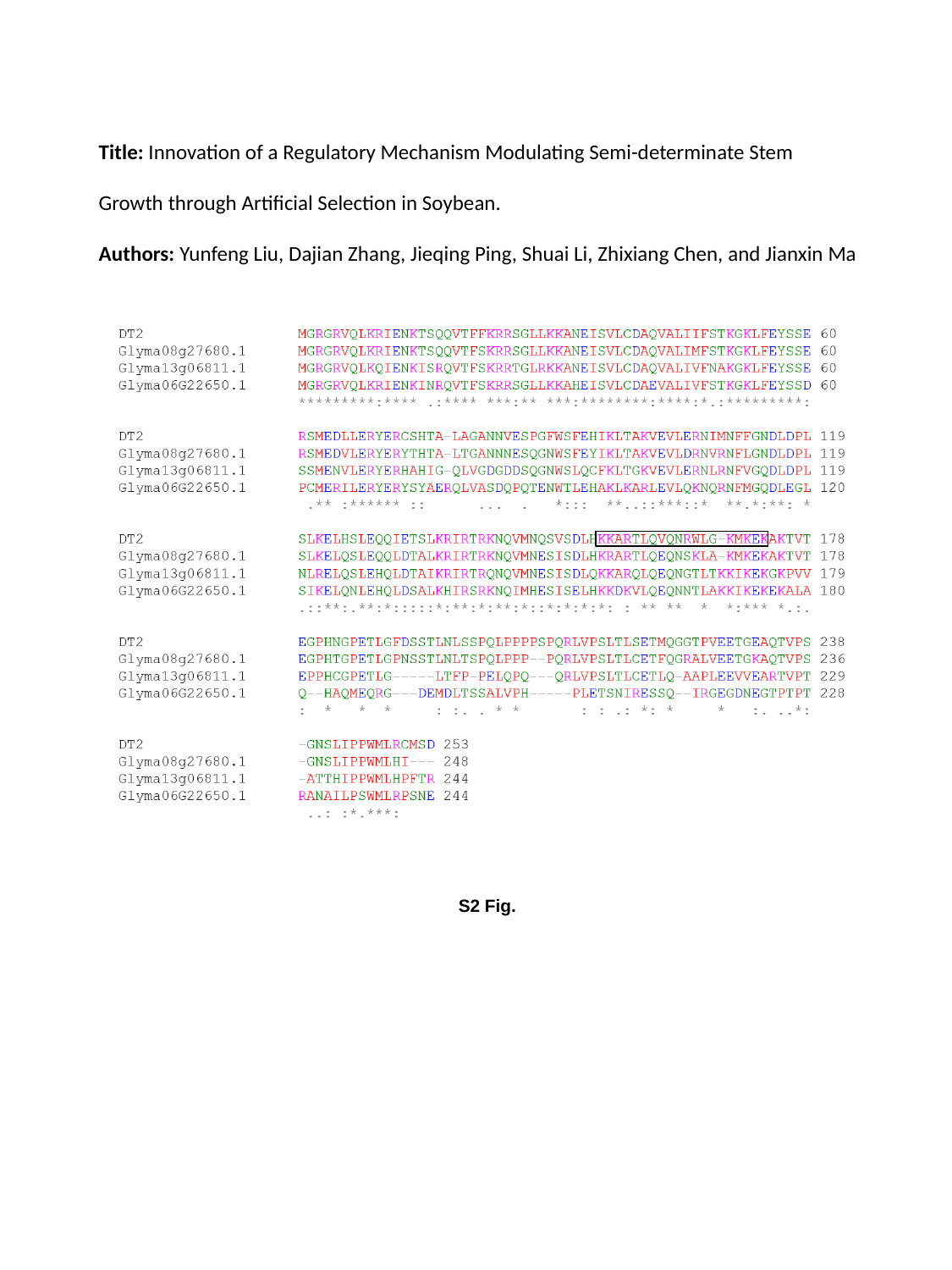

Title: Innovation of a Regulatory Mechanism Modulating Semi-determinate Stem Growth through Artificial Selection in Soybean.
Authors: Yunfeng Liu, Dajian Zhang, Jieqing Ping, Shuai Li, Zhixiang Chen, and Jianxin Ma
S2 Fig.
