# Supplementary material for: Innovation of a Regulatory Mechanism Modulating Semi-determinate Stem Growth through Artificial Selection in Soybean
Source: PLoS Genet. 2016 Jan 25;12(1):e1005818. doi: 10.1371/journal.pgen.1005818 (PMC4726468; doi:10.1371/journal.pgen.1005818)
Supplement: S1 Table — (DOCX) [file pgen.1005818.s003.docx]

**Title:** Innovation of a Regulatory Mechanism Modulating Semi-determinate Stem Growth through Artificial Selection in Soybean.

**Authors:** Yunfeng Liu, Dajian Zhang, Jieqing Ping, Shuai Li, Zhixiang Chen, and Jianxin Ma

| **S1 Table. Comparison of Genetic Interactions Underlying Stem Growth Habit Among Plants^a^** | | | | | | | | | | | | | | | | |  |
| --- | --- | --- | --- | --- | --- | --- | --- | --- | --- | --- | --- | --- | --- | --- | --- | --- | --- |
| Soybean | |  | | Arabidopsis^b^ | | |  | Tomato | | |  | | Pea | | | |  |
| Phenotype | Genotype^c^ |  | | Phenotype | | Genotype^c^ |  | Phenotype | Genotype^c^ | |  | | | Phenotype | Genotype^c^ | | |
| Semi-determinate | *Dt2Dt2;Dt1Dt1* |  | Indeterminate | | *TFL1/TFL1* | |  | Indeterminate | *SdtSdt;SPSP* |  | | Indeterminate | | | *Veg1Veg1;DetDet* | | |
| Indeterminate | *dt2dt2;Dt1Dt1* |  |  |  |  |  |  | Indeterminate | *sdtsdt;SPSP* |  | | Determinate | | | *Veg1Veg1;detdet* | | |
| Determinate | *Dt2Dt2;dt1dt1* |  | Determinate | | *tfl1/tfl1* | |  | Determinate | *SdtSdt;spsp* |  | | Never flower | | | *veg1veg1;DetDet* | | |
| Determinate | *dt2dt2;dt1dt1* |  |  |  |  |  |  | Semi-determinate | *sdtsdt;spsp* |  | | Never flower | | | *veg1veg1;detdet* | | |
| ^a^Pigeon pea and chickpea show inheritance pattern of stem growth habit and a digenic epistasis similar to observed in tomato^21, 24, 25^. | | | | | | | | | | | | | | | |  |  |
| ^b^No semi-determinacy has been observed in Arabidopsis. | | | | | | | | | | | | | | | |  |  |
| ^c^Alleles designated by names starting with capital letters are dominant over corresponding alleles written in small letters. | | | | | | | | | | | | | | | |  |  |
