# Supplementary material for: Innovation of a Regulatory Mechanism Modulating Semi-determinate Stem Growth through Artificial Selection in Soybean
Source: PLoS Genet. 2016 Jan 25;12(1):e1005818. doi: 10.1371/journal.pgen.1005818 (PMC4726468; doi:10.1371/journal.pgen.1005818)
Supplement: S2 Table — (DOCX) [file pgen.1005818.s004.docx]

**Title:** Innovation of a Regulatory Mechanism Modulating Semi-determinate Stem Growth through Artificial Selection in Soybean.

**Authors:** Yunfeng Liu, Dajian Zhang, Jieqing Ping, Shuai Li, Zhixiang Chen, and Jianxin Ma

| **S2 Table. Nine Genes Identified by Y2H Screening as Candidates encoding Proteins that Interact with Dt2.** | |
| --- | --- |
| Genes | Annotations |
| *Glyma02g05190.1* | Proteasome subunit alpha type-7-like isoform |
| *Glyma02g43040.1* | Ferritin-like domain iron ion transport |
| *Glyma02g47850.2* | Tim10/DDP family zinc finger. |
| *Glyma11g38040.1* | Molecular chaperone /Dna J |
| *Glyma12g02920.1* | Metal dependent phosphohydrolases with 'HD' motif |
| *Glyma13g34990.1* | Serine/threonine protein phosphatase |
| *Glyma17g00260.3* | Unknown protein |
| *Glyma18g45780.1 (GmSOC1)* | SOC1-like MADS-domain protein |
