# Supplementary material for: Innovation of a Regulatory Mechanism Modulating Semi-determinate Stem Growth through Artificial Selection in Soybean
Source: PLoS Genet. 2016 Jan 25;12(1):e1005818. doi: 10.1371/journal.pgen.1005818 (PMC4726468; doi:10.1371/journal.pgen.1005818)
Supplement: S3 Table — (DOCX) [file pgen.1005818.s005.docx]

**Title:** Innovation of a Regulatory Mechanism Modulating Semi-determinate Stem Growth through Artificial Selection in Soybean.

**Authors:** Yunfeng Liu, Dajian Zhang, Jieqing Ping, Shuai Li, Zhixiang Chen, and Jianxin Ma

| **S3 Table. Primers Used for PCR, RT-PCR, qRT-PCR, ChIP-PCR.** | | | |
| --- | --- | --- | --- |
| Primer Codes | Forward primer sequences (5’ to 3’) | Reverse primer sequences (5’ to 3’) | Purposes |
| Dt2-CDS | ATGGGAAGGGGTAGGGTTC | GTCGACGTCAGACATGCAG | Plasmid construction |
| Dt2-ΔC | ATGGGAAGGGGTAGGGTTC | TGGACCTTCAGTCACTGT | Plasmid construction |
| Dt2-MADS | ATGGGAAGGGGTAGGGTTC | CAGTCACTGTTGTATGGCTACATCTCT | Plasmid construction |
| Dt2-C Terminal | ATGTAGCCATACAACAGTGACTGAAGGT | GTCGACGTCAGACATGCAG | Plasmid construction |
| Dt1-Promoter | TTCTGCAGGCACACACAAATATCATT | TTGTCGACTGTAAGAGGAGGAACAAGGAAG | Plasmid construction |
| Dt1Δ-Promoter | CTGAAATGACCTCTTAGGCACTC | TTGTCGACTGTAAGAGGAGGAACAAGGAAG | Plasmid construction |
| Dt1-CDS | TTGTCGACATGGCAAAAATGCCTTTAGAG | TTTCTAGACTAGCGTCTTCTTGCAGCC | Plasmid construction |
| Dt1-Terminator | TTTCTAGATATAGCTAAGTAGCTGCTGGT | GGTACCGAGTTGTCCCAAGTTGGAGAT | Plasmid construction |
| GmSOC1-CDS | ATGGTGAGAGGAAAGACTC | GATAGACCTGGGTAGTCCA | Plasmid construction |
| Dt2-pGBKT7 | GGAATTCATGGGAAGGGGTAGGGT | GCGTCGACGTCAGACATGCAG | Y2H hybrid assay |
| GmSOC1-pGADT7 | GGAATTCATGGTGAGAGGAAAGACTC | CGAGCTCGATAGACCTGGGTAGTCCA | Y2H hybrid assay |
| Cons4-RT | GATCAGCAATTATGCACAACG | CCGCCACCATTCAGATTATGT | qRT-PCR analysis |
| Dt1-RT | GCTGGTTTTGCCACTGCAA | AAGTTATTTGAAGCCACATGTGAA | qRT-PCR analysis |
| Dt2-RT | CAGTGAGACAATGCAAGGAGGAACACCA | CCTAACTAGTCAGACATGCAGCGC | qRT-PCR analysis |
| GmSOC1-RT | GGCTGTGTGAGCAGTATGGT | ACATTGCAGTTGGGCTTCT | qRT-PCR analysis |
| Dt1-CArG1 | AATATCCAAATTTAGAAATATG | CATATTTCTAAATTTGGATATT | EMSA probe |
| Dt1-CArG2 | CTTATACCAAAATAAGCAACTAAC | GTTAGTTGCTTATTTTGGTATAAG | EMSA probe |
| Dt1-CArG3 | CCACCACCAATTAGGGCTTTTTTA | TAAAAAAGCCCTAATTGGTGGTGG | EMSA probe |
| Dt1-CArG4 | TTTCTCCAACAAAAGAATGCAAAC | GTTTGCATTCTTTTGTTGGAGAAA | EMSA probe |
| Dt1-CArG5 | TCTTTTACCAATATTGGAACTTGG | CCAAGTTCCAATATTGGTAAAAGA | EMSA probe |
| Dt1-CArG6 | GATGAGCCGAATTAAGAGACAA | TTGTCTCTTAATTCGGCTCATC | EMSA probe |
| CArG1-mutant | GAAAACCTAAAATATAAAAATTTATAAATATGAATATCAAC | GTTGATATTCATATTTATAAATTTTTATATTTTAGGTTTTC | Plasmid construction |
| CArG2-mutant | GAATATCAACTTATATTAAAATAATTAACTAACAGTTTCAGA | TCTGAAACTGTTAGTTAATTATTTTAATATAAGTTGATATTC | Plasmid construction |
| CArG5-mutant | ACCTTCCTCTTTTATTAATATTAAAACTTGGTCTGCATC | GATGCAGACCAAGTTTTAATATTAATAAAAGAGGAAGGT | Plasmid construction |
| Dt2Δc/Dt2Δk-RT | ATTTTCTACCAAAGGCAAACTTTTTG | GACATTGTTAGCTCCAGCAAGTGCTG | qRT-PCR analysis |
| SOC1-MT | CTTTTGGTTTGAACTAATCTTTGTC  GTTCACGTAGTGGGCCATC | ATATCACAAACCGTTTAGAAGCTTC | Mutant validation |
| ChIP-Dt1-P-1 | ACGAAAGAAAGTAGGAACTAAAAGA | TTGATATTCATATTTCTAAATTTGG | ChIP assay |
| ChIP-Dt1-P-2 | AATATCAACTTATACCAAAATAAGC | TTGCAGGTTGGTATCGTTTCACTGT | ChIP assay |
| ChIP-Dt1-P-3 | ACGGTCTATATATTCCACTTTC | GTACATGCACATCCCGTGCTTA | ChIP assay |
| ChIP-Dt1-P-4 | TAAGCACGGGATGTGCATGT | TGCCCTCTTCATCTCTGCATGTG | ChIP assay |
| ChIP-Dt1-P-5 | CATGCAGAGATGAAGAGGGCAA | GCCTATGGAGTATAAGCGTTTGAGTG | ChIP assay |
| ChIP-Dt1-P-6 | CAGAACTAGGATGGGATAGAAA | CACAGAATGGGGCCCTTGGAAT | ChIP assay |
| ChIP-Dt1-P-7 | AGAGGTTTGGTAGGCCATGT | GGTACTGAGTGATCGAAACCG | ChIP assay |
